# A Novel Chymotrypsin-like Protease from *Trichoderma koningii* FFT13 with Efficient Milk-Clotting Activity

**DOI:** 10.3390/foods15111940

**Published:** 2026-06-01

**Authors:** Jéssica Alves Nunes, Andreza Heloiza da Silva Gonçalves, Jeniffer Mclaine Duarte de Freitas, Josiel Santos do Nascimento, Luciano Aparecido Meireles Grillo, Melissa Fontes Landell, Hugo Juarez Vieira Pereira

**Affiliations:** 1 Institute of Chemistry and Biotechnology, Federal University of Alagoas, UFAL—Campus AC Simões, Maceió 57072-900, Brazil; 2 Institute of Pharmaceutical Sciences, Federal University of Alagoas, UFAL—Campus AC Simões, Maceió 57072-900, Brazil; 3 Institute of Biological and Health Sciences, Federal University of Alagoas, UFAL—Campus AC Simões, Maceió 57072-900, Brazil; melissa.landell@icbs.ufal.br

**Keywords:** solid-state fermentation, *Trichoderma koningii*, serine protease, milk-clotting activity, cheese production

## Abstract

Proteases, enzymes that catalyze the hydrolysis of peptide bonds in peptides and proteins, have widespread industrial applications, particularly in milk coagulation for cheese production. Microbial enzymes have been employed as alternatives to animal rennet, offering advantages such as cost-effectiveness, availability, and compliance with dietary, cultural, and religious requirements. Solid-state fermentation (SSF) is widely employed for microbial enzyme production because of its low operational costs, reduced water and energy requirements, high product concentrations, and the ability to utilize agro-industrial residues as low-cost substrates, thereby contributing to both process sustainability and waste valorization. We report the production and characterization of a novel milk-clotting protease produced by *Trichoderma koningii* FFT13. The protease was produced by SSF using wheat bran as the substrate, an agro-industrial residue. It was classified as a chymotrypsin-like serine protease and exhibited a specific caseinolytic activity of 9861 U/mg. The enzyme coagulated both reconstituted skim milk and pasteurized whole milk in the presence or absence of calcium. Coagulation was enhanced by increasing temperature, reaction time, enzyme concentration, and calcium levels. Scanning electron microscopy revealed destabilization of casein micelles, their progressive aggregation, and the formation of a well-defined gel network, confirming the effectiveness of the protease in milk coagulation. Therefore, these results demonstrate that the chymotrypsin-like protease from *T. koningii* is a promising enzyme for milk coagulation, with potential application in cheese production. The enzyme obtained constitutes an alternative to traditional coagulants, overcoming limitations related to animal rennet while potentially offering additional advantages in terms of process sustainability and industrial scalability.

## 1. Introduction

Proteases represent approximately 60% of all commercially available enzymes and are obtained from animal, plant, and microbial sources [1]. Proteases are a subgroup of hydrolases responsible for catalyzing the hydrolysis of peptide bonds in proteins, converting them into smaller fragments such as peptides and/or amino acids [2,3]. These enzymes are broadly divided into endopeptidases, which cleave peptide bonds located in the internal regions of the polypeptide chain, and exopeptidases, which act near the terminal groups of the polypeptide chain, releasing individual amino acids or di/tripeptides [4,5]. Based on their catalytic mechanism, which considers the amino acid residues present in the active site, proteases can be classified into four main subclasses: serine-, cysteine-, aspartic-proteases, and metalloproteases [6].

Serine proteases, in turn, represent the largest group of proteases and are characterized by the presence of a catalytic triad formed by histidine (His), serine (Ser), and aspartate (Asp), in which the Ser residue acts as a nucleophile in the active site, attacking the peptide bond of the substrate [7,8]. Chymotrypsin-like serine proteases have a catalytic triad composed of the amino acid residues Ser^195^, His^57^, and Asp^102^, and they perform the cleavage of peptide bonds in carbonyl groups of aromatic amino acid residues, mainly phenylalanine (Phe), tryptophan (Tpr), and tyrosine (Tyr) [9,10,11]. Proteases have applications in various industrial sectors, including the food, animal feed, dairy, pharmaceutical, and bioremediation industries [12,13].

Cheese is the most diverse and highly demanded product group in the dairy industry, with global production reaching approximately 21.3 million tons in 2020 and a market value of US$ 65–68 billion [14]. In cheese production, rennet is considered a crucial agent because it promotes milk coagulation, which is a fundamental step in developing the product’s texture. Rennet is a complex of enzymes traditionally extracted from the stomachs of unweaned calves and predominantly consists of the protease chymosin [15,16,17].

The enzymatic coagulation mechanism by chymosin involves the hydrolysis of κ-casein, specifically at the Phe^105^–Met^106^ peptide bond, producing para-κ-casein and glycomacropeptide. This cleavage compromises the stability of casein micelles, promoting their aggregation and resulting in the formation of a dense curd. Caseins (αs1, αs2, β and κ-casein) constitute 80% of the total milk proteins and are involved in curd formation, being organized into casein micelles, stabilized by calcium phosphate and hydrophobic interactions [18,19]. However, this coagulant presents disadvantages, including high cost, limited availability owing to the restricted supply of calf stomachs, and religious, ethical, and dietary concerns. Consequently, animal rennet meets only 20–30% of the global demand for milk-clotting enzymes [20].

Considering these limitations, plant and microbial proteases, for example, have been investigated as alternatives to animal rennet. Among these substitutes, proteases isolated from microorganisms have stood out due to their various advantages over coagulants of plant or animal origin, such as wide biochemical diversity and stability, possibility of genetic manipulation, and reduced production costs [21]. The growing demand also stems from the ability of the wide variety of microbial species and strains to provide proteases with diverse properties in terms of optimal temperature and pH, substrate affinity, and resistance to inhibitors [22].

Microbial coagulants are more commercially relevant for cheese production compared to coagulants of plant origin. Among the main commercial coagulants obtained from microbial sources, CHY-MAX^®^ (Novonesis, Bagsværd, Denmark), produced in *Aspergillus niger*, and coagulants derived from the fungus *Rhizomucor miehei*, such as Marzyme^®^ (IFF-DuPont, New York, NY, USA), Fromase^®^ (DSM, Maastricht, The Netherlands), Hannilase^®^ (Chr. Hansen, Hørsholm, Denmark), and Microlant^®^ (Chr. Hansen, Hørsholm, Denmark) [23]. Specifically, there has been a growing search for fungal proteases applied to milk coagulation, given the ability of various fungi to secrete these enzymes, making them the target of numerous research projects [24,25,26,27].

For enzyme production by filamentous fungi, solid-state fermentation is considered the most suitable technique because it mimics their natural growth conditions and offers advantages such as higher enzyme yields, cost-effectiveness, and the valorization and sustainable management of agro-industrial residues [28,29]. SSF is a promising approach to reduce enzyme production costs and improve the economic viability of biotechnological processes, particularly those involving enzymatic milk coagulation using proteases [30,31]. In SSF, different residues have been employed in the production of hydrolytic enzymes, including in the synthesis of proteases. These substrates are mainly derived from lignocellulosic biomass, which is predominantly composed of cellulose, hemicellulose, and lignin [32,33]. Among these residues, wheat bran has stood out as an agro-industrial residue with potential for biotechnological application in different sectors, including as a substrate for the growth of filamentous fungi for enzyme production [34].

Filamentous fungi of the genus *Trichoderma* are known for producing a wide range of enzymes, mainly hydrolases, which are used in diverse industrial processes [35]. This genus is responsible for contributing approximately 40–50% of the total value of industrial enzymes produced [36]. The fungus *Trichoderma koningii* represents an important species with the capacity to produce secondary metabolites and enzymes of industrial interest [37,38,39,40]. In the literature, the production of proteases by this species has been little investigated, and there is still a reduced number of studies involving the obtaining of these enzymes by solid-state fermentation [41].

Therefore, this study aims to evaluate the production and characterize a protease obtained from a new strain of *Trichoderma koningii*, isolated from soil samples collected in the Caatinga region of Northeast Brazil, through solid-state fermentation using wheat bran as an alternative culture medium. Specifically, the study sought to identify the enzymatic class of the protease through specific substrate assays and inhibition analysis, as well as to investigate its ability to coagulate skimmed and whole bovine milk under varying conditions of time, temperature, enzyme concentration, and calcium. This work represents the first report of a protease with milk-coagulating activity produced by *Trichoderma koningii*, expanding the biotechnological perspectives of this species and highlighting its applicability as an alternative to traditional rennet.

## 2. Materials and Methods

### 2.1. Isolation and Identification of T. koningii

The filamentous fungus *T. koningii* FFT13 was isolated from soil samples from a depth of up to 10 cm using a sterile spoon and collected at Reserva Tocaia, a conservation unit within the Caatinga biome located in Santana do Ipanema, Alagoas, Brazil. For isolation, 1 g from the soil sample was inoculated in a selective medium for cellulase-producing microorganisms composed of carboxymethylcellulose (CMC) (Sigma-Aldrich St. Louis, MO, USA) 0.5%, glucose (Dinâmica, Brazil) 0.1%, agar 2% (Kasvi, Italy), yeast extract 0.05%(Kasvi, Italy), sodium nitrate 0.1% (Vetec, Brazil), potassium phosphate 0.1% (Vetec, Brazil), potassium chloride 0.1% (Dinâmica, Brazil), magnesium sulfate 0.05% (Dinâmica, Brazil), and chloramphenicol 0.04% (Vetec, Brazil), with incubation for 7 days at 28 °C. Subsequently, 1 mL of this culture underwent two successive subcultures in the same medium and under the same incubation conditions to ensure strain purity, followed by cultivation on PDA agar (Kasvi, Italy),. Molecular identification was performed by sequencing the ITS region following the methodology described by Paulino et al. [42]. In addition, the isolate was subjected to MALDI-TOF MS analysis using a Bruker MALDI Biotyper microflex LT (Bruker Daltonics, Bremen, Germany). The strain was deposited in the culture collection of the Laboratory of Molecular Diversity and Biotechnology, Institute of Biological and Health Sciences, Federal University of Alagoas.

### 2.2. SSF of T. koningii

*T. koningii* was cultivated on potato dextrose agar and incubated in a microbiological chamber at 28 °C for 7–10 d to induce sporulation. After fungal growth, five mycelial discs (5 mm in diameter) were inoculated into 250 mL Erlenmeyer flasks containing 5 g of wheat bran with a 50% moisture content. The substrate had been previously sterilized in a vertical autoclave at 121 °C and 1.0 atm for 20 min. The wheat bran, supplied by industries located in Maceió, Alagoas, Brazil, was dried in an oven (SX 1.0 DTMC, Sterilife, Diadema, SP, Brazil) at 50 °C for 24 h and subsequently ground in a Wiley-type knife mill (ACB, Labor, São Paulo, Brazil) to obtain particles of approximately 2 mm. Fermentation was performed under static conditions in a microbiological incubator at 25 °C for 120 h.

### 2.3. Preparation of Crude Enzyme Extract (CEE) from Solid-State Fermentation

After fermentation, 5 mL of 100 mM sodium acetate buffer (pH 5.0) (Dinâmica, Brazil), was added to each gram of substrate, followed by homogenization of the mixture. The resulting suspension was filtered and centrifuged at 15,000 rpm for 1 min at 26 °C. The resulting supernatant was considered the CEE and stored in a refrigerator until further biochemical analyses.

### 2.4. Enzyme Assays

#### 2.4.1. Caseinolytic Activity

Caseinolytic activity in the CEE was determined using according to the method described by Ferreira et al. [26]. The reaction mixture consisted of 300 μL of azocasein (Sigma-Aldrich St. Louis, MO, USA) 0.6% *w*/*v* in 50 mmol/L Tris-HCl buffer, pH 8.0, 150 μL of Triton X-100 (Vetec, Brazil) 0.1% *v*/*v* in 50 mmol/L Tris-HCl buffer, pH 8.0, and 50 μL of CEE. Samples were incubated at 37 °C for 60 min. The reaction was stopped by adding 200 μL of 10% (*w*/*v*) trichloroacetic acid (Vetec, Brazil), followed by incubation at 4 °C for 30 min. Subsequently, the samples were centrifuged at 15,000 rpm for 10 min and the absorbance of the supernatant was measured at 366 nm. All assays were performed in triplicate. The reaction blank was prepared by adding TCA at the beginning of the reaction to inactivate enzymatic activity. One unit of proteolytic activity was defined as the amount of enzyme required to produce an increase of 0.01 in the absorbance at 366 nm.

#### 2.4.2. Evaluation of Proteolytic Activity for Elastase 1, Chymotrypsin, and Trypsin

The activities of elastase 1, chymotrypsin, and trypsin were determined by hydrolysis of the chromogenic substrates N-succinyl-Ala-Ala-Ala-p-nitroanilide (Sigma-Aldrich St. Louis, MO, USA), N-succinyl-Ala-Ala-Pro-Phe-p-nitroanilide (Sigma-Aldrich St. Louis, MO, USA), and benzoyl-arginine-p-nitroanilide (Sigma-Aldrich St. Louis, MO, USA), respectively, according to Ferreira et al. [11]. Stock solutions of these substrates were prepared in DMSO (Vetec, Brazil) at a concentration of 50 mmol/L. For the enzymatic assays, 30 μL of substrate (previously diluted in 50 mmol/L Tris-HCl buffer, pH 8.0, to a final concentration of 0.25 mmol/L) was added to 110 μL of the same dilution buffer and 10 μL of CEE. The reaction mixtures were incubated at 37 °C for 1 h, and the release of p-nitroanilide was measured at 410 nm. Reaction blanks were prepared without CEE. All assays were conducted in triplicate. Enzymatic activity was calculated using the following equation:

$$AE\left(U{mL}^{-1}\right)=\frac{\left(A-A_{0}\right)\times Fv\left(mL\right)\times 1000}{8800\times T(min)\times 0.2}$$

where EA = enzymatic activity; A and A_0_ = absorbance values measured at 410 nm for the sample and blank, respectively; Fv = final volume; T = reaction time; and 8800 M/cm = molar extinction coefficient of p-nitroanilide.

#### 2.4.3. Determination of the Effect of Inhibitors on Enzymatic Activities

The effect of protease inhibitors on enzymatic activity was evaluated using phenylmethylsulfonyl fluoride (Sigma-Aldrich St. Louis, MO, USA) 5 mmol/L, EDTA (Sigma-Aldrich St. Louis, MO, USA), β-mercaptoethanol (Sigma-Aldrich St. Louis, MO, USA) and benzamidine (Sigma-Aldrich St. Louis, MO, USA) 1 mmol/L. The inhibitors were preincubated with the enzyme for 30 min prior to substrate addition. After preincubation, the substrate was added to the samples and the enzyme assays were performed as described in Section 2.4.1 and Section 2.4.2. The assays were performed as previously described by our research group [43]. The control consisted of enzymatic activity measured in the absence of any inhibitor and was defined as 100% enzymatic activity. The percentage of inhibition was calculated based on the reduction in enzymatic activity in the presence of inhibitors, according to the following equation:

Inhibition (%) = [(Control activity − Activity with inhibitor)/Control activity] × 100.

#### 2.4.4. Zymography

Proteolytic activity was evaluated by zymography using a polyacrylamide gel copolymerized with casein as the substrate under native electrophoretic conditions [44]. An 8% polyacrylamide gel containing casein at a final concentration of 4% (*w*/*v*) was prepared. Samples were prepared by mixing 50 μL of CEE with 5 μL of sample buffer composed of 0.5 M Tris-HCl (pH 6.8), SDS (2%), glycerol (10%), and bromophenol blue (0.001%) (Sigma-Aldrich St. Louis, MO, USA). Electrophoresis was performed at a constant voltage of 120 V (Bio-Rad Laboratories Hercules, CA, USA). After electrophoresis, the gel was washed twice in 0.5 M Tris-HCl buffer (pH 8.0) containing 2.5% Triton X-100 for 15 min each. Subsequently, the gel was incubated in 0.5 M Tris-HCl buffer (pH 8.0) at 37 °C for 48 h. Following incubation, the gel was stained with Coomassie Brilliant Blue G-250 (Sigma-Aldrich St. Louis, MO, USA) and destained in a solution containing 40% methanol, 10% acetic acid, and 50% water.

#### 2.4.5. Determination of Protein Concentration

Protein concentration was determined according to the Bradford method [45], using bovine serum albumin as a standard. A 10 μL aliquot of sample was added to 190 μL of Bradford reagent (Sigma-Aldrich St. Louis, MO, USA), followed by incubation for 5 min. Absorbance was measured at 595 nm, and protein concentration expressed in mg/mL.

### 2.5. Application of Protease in Milk Coagulation and Study of Coagulation Conditions

#### 2.5.1. Coagulation and Milk-Clotting Activity Assays

Coagulation assays were performed using reconstituted skim milk (RSM) and pasteurized whole milk (PWM), as described by Cavalcante [46]. All analyses were conducted in triplicate, with extraction buffer used instead of the CEE in the control reactions. MCA was determined based on the amount of enzyme required to coagulate 10 mL of milk within 40 min under the established assay conditions. MCA was calculated using the following equation:

MCA (U/mL) = [(2400 × V)/t] × v

where “V” = volume of milk (mL), “t” = coagulation time (s), and “v” = volume of enzymatic extract used (mL).

To determine the minimum protein concentration required for milk coagulation, a concentration curve was generated using CEE at 0.202, 0.101, 0.067, 0.050, and 0.033 mg/mL, diluted in 100 mM sodium acetate buffer (pH 5.0). Coagulation assays were performed by adding 100 μL of each dilution to 500 μL of RSM and incubating at 37 °C for 120 min. The effects of time and temperature on maximum observed coagulation activity were evaluated using RSM (10% *w*/*v* in 10 mM CaCl_2_) at 25, 37, and 50 °C, with incubation periods ranging from 5–100 min. Assays were conducted in triplicate with 16.6 μL of CEE and 250 μL of milk, while control samples contained buffer instead of enzyme. Clot formation was assessed after centrifugation at 2000× *g* for 2 min. The whey protein profile from samples incubated at 37 °C was analyzed by 15% SDS-PAGE following Laemmli’s method, with Coomassie Brilliant Blue G-250 staining. To assess thermal stability, CEE samples with and without prior heating at 100 °C for 15 min were incubated at 37 °C for 4 h, followed by centrifugation to verify coagulation.

#### 2.5.2. Microstructure Analysis by Scanning Electron Microscopy (SEM)

Microstructural changes during enzymatic milk coagulation were analyzed by SEM. Samples were collected from the control (without enzyme addition) and at different stages of the coagulation process (5, 15, 20, 40, 60, 80, and 100 min). For analysis, the samples were mounted on stubs and coated with a thin layer of gold (approximately 20–30 nm thick) by sputter deposition using an SSX-550 Superscan microscope (Shimadzu, Kyoto, Japan).

#### 2.5.3. Effect of Calcium Concentration and PWM on Enzymatic Milk Coagulation

RSM coagulation assays were performed using different calcium concentrations in the reaction medium (0, 0.1, 0.25, 0.5, 2, 4, 6, 8, and 10 mmol/L). In each assay, 16.6 μL of CEE was added and the reactions were incubated for 120 min at 25, 37, and 50 °C, according to the procedures described in Section 2.4.1. The MCA of the CEE was also evaluated using PWM, following the same procedures described for RSM. Reactions were conducted in the presence or absence of calcium. In the presence of calcium (10 mM), samples were incubated until complete clot formation, and the coagulation time was recorded. In the absence of calcium, clot formation was monitored at 60 and 120 min intervals. All assays were performed in triplicate with their respective controls. At the end of the incubation period, samples were centrifuged to assess clot formation.

### 2.6. Statistical Analyses

All experiments were performed in triplicate, and the results were expressed as mean ± standard deviation. Data analysis and graph construction were performed using Origin software (version 8.0).

## 3. Results and Discussion

### 3.1. Verification of the Caseinolytic Activity of the CEE

*T. koningii* was subjected to SSF using wheat bran as the substrate, and enzyme production was monitored over time, with the highest activity observed at 120 h of cultivation. The CEE obtained under these conditions exhibited a specific caseinolytic activity of 9861 U/mg against the azocasein substrate. These values were higher than those reported for proteases present in the CEEs of *Pycnoporus sanguineus* [26], *Aspergillus oryzae* Y1 [47], and *Pleurotus sajor-caju* CTM10057 [48], which exhibited specific proteolytic activities against azocasein of 741.3, 226.9 and 8823 U/mg, respectively.

**Figure 1:**
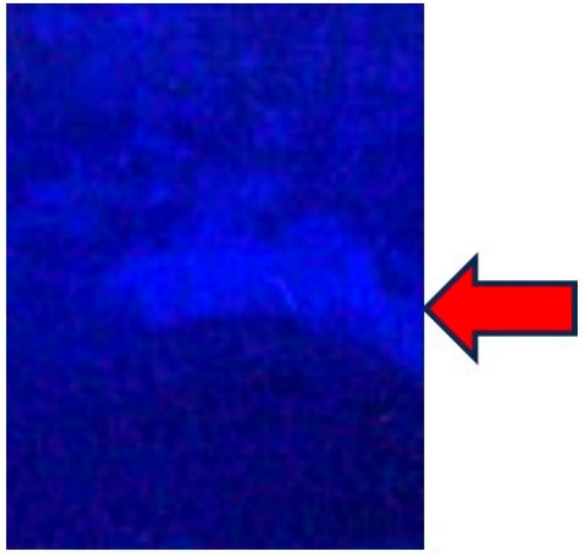
Casein zymography. The clear areas observed against the blue background correspond to zones of casein degradation (red arrow).

Zymogram analysis revealed casein hydrolysis, evidenced by clear zones against a Coomassie blue-stained casein background (Figure 1), confirming the action of proteases in the enzymatic extract and their ability to maintain catalytic activity under solid medium conditions. Proteolytic activity has also been confirmed by casein zymography for a novel serine protease produced by *Mucor subtilissimus* URM 4133 [49] and a metalloprotease from *Termitomyces clypeatus* MTCC 5091 [50]. Given that casein is the primary substrate involved in milk coagulation, casein zymography is particularly relevant, as it directly demonstrates the ability of the enzyme to hydrolyze this protein complex, thereby providing experimental evidence of its potential application as a milk-clotting agent in the dairy industry.

### 3.2. Effect of Inhibitors

The CEE was evaluated against different synthetic substrates specific for elastase 1, chymotrypsin, and trypsin-type proteases. Among the substrates tested, enzymatic activity was detected exclusively for N-succinyl-Ala-Ala-Pro-Phe-p-nitroanilide (8.72 × 10^−1^ mU/mL), a substrate specific for chymotrypsin, while no activity was observed toward the other substrates. The absence of activity against substrates specific for trypsin and elastase 1, combined with the exclusive detection of activity toward the chromogenic substrate specific for chymotrypsin, indicates that the CEE does not contain trypsin-like or elastase I-like serine proteases but instead exhibits chymotrypsin-like activity.

Subsequently, the effects of different inhibitors on caseinolytic and chymotrypsin-like activities were assessed, and the results were expressed as percentage inhibition (Table 1). Inhibition assays were performed using specific inhibitors of serine, cysteine, and metalloproteases, as well as the trypsin-specific inhibitor benzamidine, to further characterize the catalytic class of the protease under study (Table 1). EDTA (a metalloprotease inhibitor), benzamidine (a trypsin inhibitor), and β-mercaptoethanol (a cysteine protease inhibitor) had little to no effect on the measured activities (Table 1). In contrast, PMSF, an irreversible serine protease inhibitor, exhibited the highest percentage inhibition of both substrates, causing 84.17% inhibition of caseinolytic activity and 100% inhibition of chymotrypsin-like activity. These results confirmed the involvement of a serine residue in the catalytic mechanism of the chymotrypsin-like caseinolytic protease. PMSF inhibits serine protease irreversibly by sulfonating the serine residue at the enzyme’s active site, thereby blocking catalytic activity [51]. Supporting these findings, a chymotrypsin-like serine protease from *Pycnoporus sanguineus* has also been reported to inhibit caseinolytic activity by 62.59% and completely suppress chymotryptic activity in the presence of PMSF [26]. Inhibition by PMSF has also been reported for proteases produced by other filamentous fungal species, such as *Trichoderma longibrachiatum* [52], *Aspergillus oryzae* [51], and *Mucor subtilissimus*, confirming the involvement of a serine residue in the active site [49].

### 3.3. Application of Chymotrypsin-like Enzyme in Milk Coagulation and Evaluation of Coagulation Conditions

#### 3.3.1. Effect of Protein Concentration in CEE

**Figure 2:**
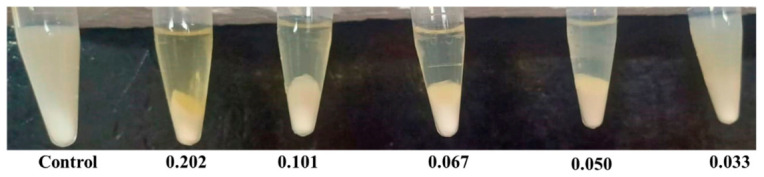
Dose–response effect of the CEE concentration (mg·mL^−1^) on the coagulation of reconstituted skim milk (RSM) at 37 °C for 120 min.

The CEE was evaluated for RSM coagulation at five protein concentrations: 0.202, 0.101, 0.067, 0.050, and 0.033 mg/mL (Figure 2). The concentration-response curve showed that the protease in the CEE was capable of inducing coagulation at protein concentrations as low as 0.050 mg/mL. Coagulation time decreased as the protein concentration increased (Figure 2).

For subsequent milk coagulation experiments, a protein concentration of 0.067 mg/mL was selected, as it represented the lowest concentration capable of achieving coagulation within a considerably shorter time than that of 0.050 mg/mL. Several factors may influence the milk coagulation process, including temperature, pH, Ca^2+^ concentration, fat content, and enzyme dosage. Increasing the enzyme concentration accelerates the enzymatic phase of milk coagulation [15,53]. Ferreira et al. [26] reported clot formation starting at 10.4 mg mL^−1^, with increased coagulation as a function of increasing protein concentration.

#### 3.3.2. Coagulation Time and Temperature Using RSM

The CEE of *T. koningii* (0.067 mg/mL) successfully coagulated RSM at various temperatures. Clot formation occurred within 30 min at 37 °C, and this time was halved when the temperature was increased to 50 °C. Chinmayee et al. [54] reported increased curd yield when applying enzymatic extracts from *Mucor thermohyalospora* and *Rhizopus azygosporus* as the temperature increased from 37 to 45 °C, supporting that microbial coagulants exhibit maximum activity at temperatures approaching 50 °C. Table 2 presents a comparison of the maximum coagulation temperature for different enzyme sources described in the literature, including enzymes of fungal and bacterial origin.

At room temperature (25 °C), coagulation was observed) after 100 min, representing an advantageous feature for industrial applications as it eliminates the need for strict thermal control and reduces associated processing costs.

Furthermore, heating the CEE at 100 °C for 15 min completely abolished its ability to coagulate the RSM. This confirms that the coagulant activity *T. koningii* CEE is directly attributable to proteases, as enzymes undergo thermal denaturation at high temperatures, resulting in conformational changes in their three-dimensional structures that eliminate their catalytic activity [57].

### 3.4. Protein Profile of Whey After Proteolysis by CEE

**Figure 3:**
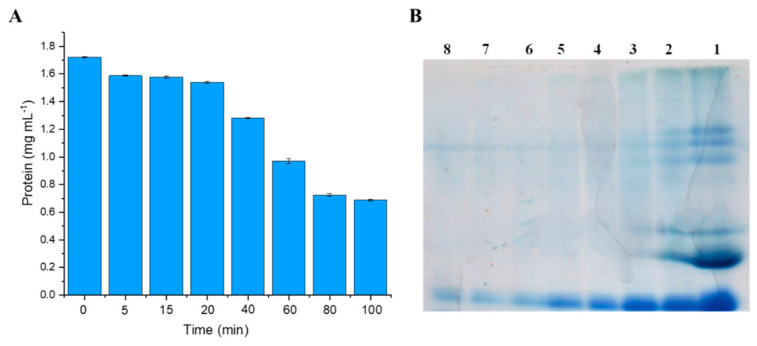
Serum protein profile of samples used in the RSM coagulation time study at 37 °C. (**A**) Protein concentration of supernatants determined by the Bradford method. (**B**) Electrophoresis of samples at different incubation times (1—reaction blank; 2—5 min; 3—15 min; 4—20 min; 5—40 min; 6—60 min; 7—80 min; 8—100 min).

During incubation (5–100 min), whey protein concentration gradually decreased, consistent with the Bradford protein concentration curve (Figure 3A). A pronounced decline in protein concentration occurred from 40 min onward, after which the band pattern became relatively stable, remaining similar at 60, 80, and 100 min (Figure 3B).

During enzymatic coagulation, proteases hydrolyze-specific peptide bonds in κ-casein, thereby reducing the repulsive forces between micelles and consequently promoting their aggregation and clot formation after approximately 70% cleavage. Consequently, the protein content of whey progressively decreased (Figure 3A,B) as micelles aggregated, facilitating curd consolidation [58].

SDS-PAGE analysis of whey samples at 37 °C revealed the protein bands at different incubation times (Figure 3B). The control sample (lane 1) showed a higher total protein content, as indicated by more intense and well-defined bands. The bands became less intense as incubation time increased, reflecting ongoing proteolysis and curd formation.

### 3.5. Structural Characterization of the Formed Clot by SEM

**Figure 4:**
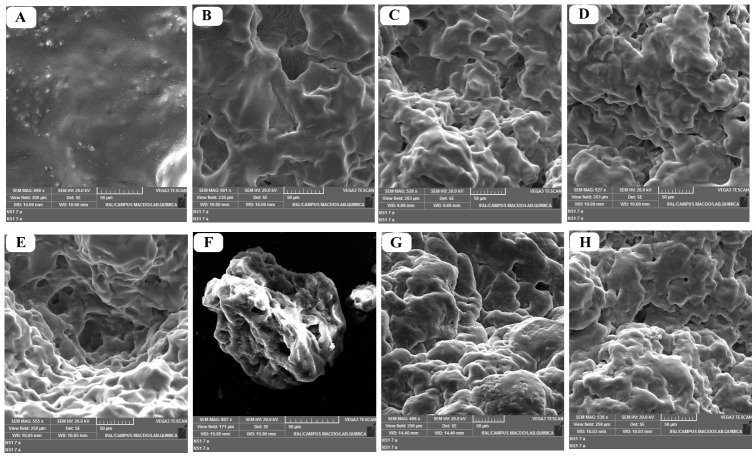
Scanning electron micrographs of milk during coagulation at different reaction times (scale bar = 50 µm). (**A**) Control; (**B**) 5 min; (**C**) 15 min; (**D**) 20 min; (**E**) 40 min; (**F**) 60 min; (**G**) 80 min; (**H**) 100 min.

Clot formation during enzymatic milk coagulation was analyzed by SEM, which allowed the observation of the progression of casein micelle aggregation (Figure 4). In the control micrograph (Figure 4A), in which no enzyme was added to the milk, a smooth and homogeneous surface was observed, indicating the absence of aggregation. This reflects the preservation of milk colloidal stability owing to the lack of κ-casein hydrolysis.

In the presence of the enzyme, at the initial reaction times, the microstructures exhibited surface roughness and the onset of micelle contact, evidencing the destabilization of these colloidal structures owing to κ-casein hydrolysis (Figure 4B–D). Aggregation of para-micelles occurred, which was more clearly visualized in the micrograph shown in Figure 4E, corresponding to 40 min of the reaction, where more pronounced surface roughness was observed.

As coagulation progressed, the micrographs revealed a highly interconnected structure with increased compactness and uniformity, characteristic of the gel formation phase (Figure 4F–H). These observations were consistent with those reported in other studies that employed SEM to analyze the microstructure of milk gels formed by enzymatic coagulation [19,59,60]. Chen et al. [61], using SEM to analyze the microstructure of curds produced with proteases from *Bacillus subtilis* and *Rhizopus oligosporus*, in comparison with commercial rennet, observed that a denser and smoother network, with fewer granules, is associated with the formation of a higher quality curd.

### 3.6. Effect of Calcium Concentration on Milk Coagulation

**Figure 5:**
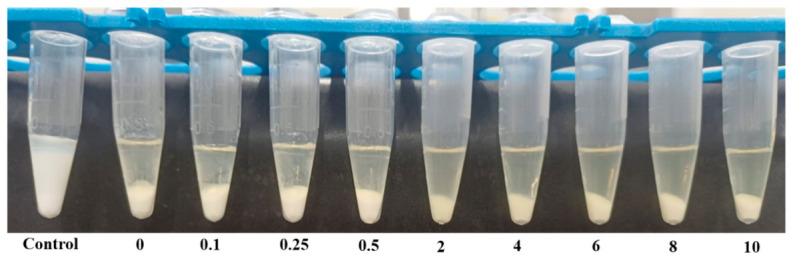
Coagulation of RSM at 50 °C with Ca^2+^ concentrations ranging from 0–10 mmol/L.

The effect of calcium concentration was evaluated using the lowest protein concentration (0.067 mg/mL) in the CEE capable of coagulating RSM at three temperatures (25, 37, and 50 °C) over 120 min (Figure 5). At 25 and 37 °C, coagulation occurred only at the highest calcium concentrations tested, up to 8 mmol/L at 25 °C and 4 mmol/L at 37 °C (Figure 5). In contrast, at the maximum temperature of 50 °C, coagulation occurred at all calcium concentrations evaluated, including in the absence of CaCl_2_. Increasing calcium supplementation in the reaction medium reduced coagulation time, as evidenced by the greater transparency of the supernatants (Figure 5). These results indicate that the protease from *T. koningii* exhibits enhanced coagulant activity with increasing calcium concentration and elevated temperature, corroborating the findings of the previous section, where a marked reduction in coagulation time was observed at 50 °C.

The observed behavior is consistent with reports on proteases from *Aureobasidium leucospermi* LB86 [62] and *Rhizopus microsporus* [63] produced by SSF, where increased calcium concentration resulted in enhanced coagulant activity. Calcium chloride is widely used in milk coagulation because it reduces gelation time, enhances curd texture and yield, and significantly decreases protein hydrolysis and bitterness [64,65]. The addition of Ca^2+^ neutralizes electrostatic repulsion between casein micelles and indirectly promotes a slight reduction in milk pH through the precipitation of soluble calcium and phosphate as colloidal calcium phosphate, accompanied by the release of H^+^ into the medium, thereby favoring micelle aggregation [66,67]. In addition to its effect on the substrate, Ca^2+^ may act as an enzymatic catalytic agent [68].

The protease present in the CEE was further evaluated in coagulation assays using PWM, both in the absence and presence of calcium, at 25, 37, and 50 °C. Coagulation was observed under all the tested reaction conditions. Whole milk has higher fat content than skim milk, which plays a crucial role in the textural, functional, and sensory properties of dairy products [43]. Similarly, Aljammas et al. [68] reported higher coagulation activity in whole milk than in skim milk when using the proteases Rm4 and MW150 from *Rhizomucor miehei* as coagulants. This effect was attributed to the hydrophobic interactions promoted by milk fat, which facilitate protease access to peptide bonds in casein. These aspects, particularly the effects on cheese yield, texture, and sensory properties, should be further evaluated in future studies to better assess the enzyme’s industrial applicability.

## 4. Conclusions

In this study, the CEE produced by *T. koningii* FFT13 during SSF using wheat bran exhibited a specific caseinolytic activity of 9861 U/mg and also showed activity toward a chymotrypsin-specific substrate, exhibiting a value of 8.72 × 10^−1^ mU/mL. The strong inhibition of both caseinolytic and chymotrypsin-like activities in the presence of PMSF led indicates that the coagulant protease from *T. koningii* is a chymotrypsin-like serine protease. This enzyme coagulated both RSM and PWM in the presence or absence of calcium. At a protein concentration of 0.067 mg·mL^−1^, the maximum coagulation temperature was observed at 50 °C. The different conditions evaluated, including reaction time, temperature, enzyme concentration, and presence of calcium ions, influenced milk coagulation, which was enhanced by increasing any of these factors. SEM analysis confirmed that the protease promotes κ-casein hydrolysis, thereby triggering micelle destabilization, subsequent micelle aggregation, and the formation of a three-dimensional interconnected and compact network characteristic of a gel. These findings demonstrated the enzyme’s effectiveness in milk coagulation and suggest its potential to produce curds with structural characteristics compatible with cheese production. To our knowledge, this is the first report on a milk-coagulating chymotrypsin-like serine protease produced by *T. koningii*. These findings indicate that the protease produced by *T. koningii* FFT13 is a promising and economically viable alternative for applications in the dairy industry, particularly as a coagulant in cheese sustainable production. Future research directions will focus on detailed biochemical characterization of the protease, as well as evaluating the enzyme’s performance in real cheese-making processes to assess its impact on yield, texture, and sensory properties, aiming for its industrial applicability.

## Figures and Tables

**Table 1:** Percentage of inhibition of caseinolytic and chymotrypsin-like activities in the presence of different protease inhibitors.

Inhibitor	Inhibition of Caseinolytic Activity (%)	Inhibition of Chymotrypsin-like Activity (%)
PMSF	84.17 ± 1.17	100.00 ± 0.47
β-mercaptoethanol	18.30 ± 2.49	3.50 ± 0.45
EDTA	0.00 ± 4.98	1.60 ± 3.57
Benzamidine	0.00 ± 5.48	5.40 ± 1.34

Abbreviations: PMSF—phenylmethylsulfonyl fluoride, EDTA—ethylenediaminetetraacetic acid.

**Table 2:** Comparison of milk-clotting enzymes from different microbial sources, including enzyme class, optimal temperature, and coagulation time.

Study	Enzymatic Source	Species	Protease Class	Maximum Coagulation Temperature (°C)	Coagulation Time (min)
This study	Fungus	*Trichoderma koningii*	Serine protease	50	15
[26]	Fungus	*Pycnoporus sanguineus*	Serine protease	50	120
[43]	Fungus	*Pleurotus djamor*	Serine protease	50	45
[55]	Fungus	*Pleurotus florida*	–	55	–
[56]	Bacterium	*Pediococcus acidilactici* SH	–	50	–

## Data Availability

All relevant data are within the paper and in the references.

## Abbreviations

The following abbreviations are used in this manuscript:

CEE	Crude enzyme extract
DMSO	Dimethyl sulfoxide
EDTA	Ethylenediaminetetraacetic acid
MCA	Milk-clotting activity
PMSF	Phenylmethylsulfonyl fluoride
PWM	Pasteurised whole milk
RSM	Reconstituted skim milk
SDS	Sodium dodecyl sulfate
SDS-PAGE	Sodium dodecyl sulfate–polyacrylamide gel electrophoresis
SEM	Scanning electron microscopy
SSF	Solid-state fermentation
TCA	Trichloroacetic acid
